# Population-attributable fractions for risk factors for childhood anaemia: findings from the 2017 Togo Malaria Indicator Survey

**DOI:** 10.1017/S0007114524003313

**Published:** 2025-11-14

**Authors:** Phyllis Ohene-Agyei, Aude Laetitia Ndoadoumgue, Essossimna Bana-Ewai, Issifou Yaya, Aboubakari Nambiema

**Affiliations:** 1 Unité de Recherche en Santé des Populations (URESAP), Centre Hospitalier Universitaire Sylvanus OLYMPIO (CHU SO), Lomé, Togo; 2 University of Ghana, Legon-Accra, Ghana; 3 Association de la Diaspora pour le Recherche et la Promotion de la Santé en Afrique (ADREPSA), Angers, France; 4 Occupational Health Office, TOGOCOM in Togo, Lomé, Togo; 5 Société Togolaise de Médecine du Travail (STMT), Lomé, Togo; 6 Unité de Recherche Clinique en Economie de la Santé (URC-ECO), Hôpital Hôtel-Dieu, Assistance Publique Hôpital de Paris, Paris, France; 7 Université Paris Cité, INSERM U970, Paris Cardiovascular Research Centre (PARCC), Integrative Epidemiology of Cardiovascular Disease, Paris, France

**Keywords:** Children 6–59 months, Anaemia, Associated factor, Population-attributable fraction, Cross-sectional study, Togo

## Abstract

Anaemia remains a significant public health concern in developing countries. This study estimated the proportion of childhood anaemia cases that could be potentially prevented in Togo using data from the 2017 National Malaria Indicator Survey. Maternal, child, and household data were collected through standardized face-to-face interviews. Haemoglobin (Hb) levels were measured in children and their mothers. A total of 2796 children were included in the analyses. The prevalence of anaemia was 75·0 % (95 % CI, CI: 72·5, 88·0). Factors associated with childhood anaemia were age ((adjusted prevalence ratio, aPR = 1·46 (CI: 1·37, 1·56) for 6–23 months and aPR = 1·23 (1·14, 1·32) for 24–42 months, ref: 43–59 months), a later birth order (≥ 4th position) (aPR = 1·11 (1·03, 1·19), ref: 1st–2nd position), malaria in children (aPR = 1·30 (1·22, 1·38)), maternal age ≤ 25 (aPR = 1·17 (1·08, 1·27), ref: ≥ 35 years), maternal anaemia (aPR = 1·13 (1·07, 1·19)), lack of maternal education (aPR = 1·10 (1·02, 1·18), ref: ≥ secondary), number of children under 5 in household (aPR = 1·07 (1·00, 1·14) for ≥ 3, ref: 0–1), unimproved sanitation facilities (aPR = 1·12 (1·02, 1·22)) and low/middle household incomes (aPR = 1·16 (1·04, 1·30) and aPR = 1·13 (1·01, 1·26), respectively, ref: high). The population-attributable fraction was estimated at 8·2 % (6·3, 10·1 %) for child-related modifiable factors, 11·1 % (5·7, 16·3 %) for maternal-related factors, 15·8 % (8·6, 22·5 %) for household-related factors and 30·9 % (24·0, 37·2 %) for the combination of all modifiable factors. This study highlighted a high prevalence of childhood anaemia in Togo and showed that a high proportion of this could be prevented.

Anaemia is a public health concern that affects different demographics in both developed and developing countries^(1)^. Globally, children under 5 years of age are a group with a high burden of anaemia^(2)^. Further disaggregation by region reveals that sub-Saharan Africa (SSA) has the highest prevalence of anaemia in children under 5 years, which was estimated at 68 %^(3)^.

The causes of childhood anaemia are multifactorial and include nutritional deficiencies (particularly iron deficiency), infections and blood disorders^(4–6)^. Anaemia in children not only has consequences, which are not just limited to health (cognitive function impairment, decreased immune function that exposes children to infections, etc.), but also has adverse social and economic effects (e.g. performance in school, physical and behavioural growth, reduced productivity in adult life). Studies have shown that childhood iron deficiency anaemia impairs immune function resulting in increased morbidity, poor motor and cognitive development and social and emotional development^(7–11)^. A recent pooled study^(12)^ including thirty-two cross-sectional studies conducted in SSA countries found that the prevalence of childhood anaemia ranged from 36·6 % in Rwanda to 88·0 % in Burkina Faso. Furthermore, this study also revealed diverse risk factors for childhood anaemia including demographic, socio-economic factors, family structure, maternal health, child nutrition and recent episodes of illnesses. In 2019, the prevalence of childhood anaemia in Togo was reported at 72·4 % compared with 79·8 % two decades ago^(13)^. However, there exist few population-based studies on childhood anaemia in Togo. One of such studies conducted by Nambiema *et al.* ^(14)^, using data from Togo’s 2013–2014 Demographic and Health Surveys (DHS), reported a high prevalence of anaemia estimated at 70·9 % and examined the factors potentially associated with childhood anaemia but did not quantify the proportion of anaemia that could be attributable to associated factors.

Furthermore, with regard to population-based disease prevention, interventions should consider the risk factors that contribute most to the occurrence of the disease within a population. To assess the effect of an exposure factor on the occurrence of disease at the population level, an alternative to conventional estimates (e.g. relative risk, odd ratio, prevalence ratio) would be to quantify the proportion of cases of disease attributable to that factor by the computation of the population-attributable fraction (PAF), also known as population-attributable risk. The concept of PAF has found widespread application in public health epidemiological studies^(15–17)^ including in SSA^(17)^. Moschovis *et al.* ^(17)^ estimated the PAF of childhood anaemia using aggregate DHS data of twenty-seven SSA countries. However, these valuable findings were based on aggregate data and do not allow for the implementation of local prevention strategies that consider local realities.

This paper sought to estimate the proportion of childhood anaemia cases that could be attributable to child-related, maternal and household factors among Togolese children aged 6–59 months, using data from the 2017 Togo Malaria Indicator Survey (2017 TMIS), the last one conducted to date. This survey is the only one in Togo that provides nationally representative data. The decision to use PAF calculations is to help quantify the burden of childhood anaemia, which could be reduced by focusing on potentially modifiable factors. This could ultimately contribute to estimating the impact and cost-effectiveness of anaemia interventions in Togo.

## Methods

### Data source, study design and population

This study analysed the data from the 2017 TMIS, which was conducted between September and November 2017 and included 3669 children born in the last 5 years. The 2017 TMIS is a national cross-sectional survey designed to obtain population-based estimates of malaria indicators to complement routine administrative data that are used to direct strategic planning and evaluation of Togo’s malaria control programme. Maternal, child and household data were collected using a standard questionnaire administered face-to-face by trained interviewers. In addition, haemoglobin (Hb) tests were conducted for children aged 6–59 months and their mothers. Capillary Hb testing was performed with the HemoCue Photometer, a device commonly used in screening for anaemia in low-resource settings, which produces a result in less than a minute^(18)^. The indicators’ estimates were for the country as a whole, urban and rural areas separately, and each of the five administrative regions (Savanes, Kara, Centrale, Plateaux and Maritime without Lomé) and the agglomeration of Lomé. More information about the survey can be found in the final report^(19)^. Data from the 2017 TMIS are freely accessible on the Malaria Indicator Survey and the DHS programme websites via https://malariasurveys.org/index.cfm and https://www.dhsprogram.com/, respectively.

### Definition of variables

The main outcome variable in this study was anaemia, classified using the WHO’s thresholds based on Hb concentrations^(1)^. In children aged 6–59 months, anaemia was defined as a reduced level of Hb in the blood less than 110 g/L.

Independent variables were selected from the 2017 TMIS data based on potential association with risk of childhood anaemia and categorised under three groups as follows:

(1) Child-related factors: sex (male/female), age in months (categorised: ‘6–23’, ‘24–42’, ‘43–59’), birth order (order number of the births from first to last: ‘1–2’, ‘3’ and ≥ 4). Malnutrition (yes/no); malaria infection (yes/no) was defined from a diagnosis based on both a rapid diagnostic test and a blood smear test and history of fever (2 weeks preceding the survey) (yes/no).

(2) Maternal factors: age in years (categorised: ≤ 25, ‘26–34’ and ≥ 35), anaemia (yes/no) defined as Hb < 110 g/L for everyone except pregnant women and Hb < 120 g/L for pregnant women, pregnancy status (yes/no) (whether the respondent was currently pregnant at the time of the interview), educational level (categorised as no education, primary and secondary/higher).

(3) Household factors: sex of the household head (male/female), age of the household head (categorised: ≤ 32, ‘33–48’ and ≥ 49), number of children 5 and under in household (categorised as follows: ‘0–1’, 2 and ≥ 3), unimproved drinking water source (yes/no), type of sanitation facilities (categorised as follows: ‘unimproved sanitation facilities’, ‘shared sanitation facilities’ and ‘improved sanitation facilities’), place of residence (rural/urban) and region of residence (including the five administrative regions: Savanes, Kara, Centrale, Plateaux and Maritime without Lomé and agglomeration of Lomé), income status (grouped into three categories based on the standardised variable constructed by the DHS using permanent income indicators ‘low (1st and 2nd quintiles)’, ‘middle (3rd quintile)’ and ‘high (4th and 5th quintiles)’^(20)^. The income status indicator was defined using the wealth index, a composite measure of a household’s cumulative standard of living, calculated from data on the household’s ownership of selected assets. Following WHO guidelines^(21)^, ‘unimproved sanitation facilities’ were defined as pit latrines without a slab or platform, hanging latrines, bucket latrines and open defaecation (no toilet); ‘shared sanitation facilities’ were defined as sanitation facilities of an otherwise acceptable type shared between two or more households; and ‘improved sanitation facilities’ (not shared or not public) include flush/pour flush to piped sewer system septic tank or pit latrine, ventilated improved pit latrine, pit latrine with slab and composting toilet. Following the same previous WHO guidelines, an ‘improved drinking water source’ was defined as the main source of drinking water of piped household water connection located inside the user’s dwelling, plot or yard, public taps or standpipes, tube wells or boreholes, protected dug wells, protected springs or rainwater collection. All other sources were considered unimproved.

### Statistical analysis

All statistical analyses were performed using Stata/se V.15. Both weighted and unweighted (crude) prevalence of childhood anaemia were estimated in descriptive analyses. In weighted prevalence, survey weights were used to account for the unequal probability of selection of households induced by the sampling scheme as well as for non-response. A detailed explanation of the weighting procedure can be found in the DHS Methodology report^(22)^. According to the final report^(19)^, the 2017 TMIS results were representative of the whole country.

#### Assessment of associated factors and population-attributable fraction estimate

To examine associations between factors and childhood anaemia, bivariate and multivariable Poisson regression models with robust variance^(23)^ were performed using Stata/SE V.15 survey procedures to estimate crude (cPR) and adjusted prevalence ratios (aPR) and their 95 % CI. The *svy* procedures in Stata/SE V.15 are a set of commands that consider sampling weights, clustering and stratification in complex survey data. Only factors with a Wald test *P*-value of less than 0·20 in the bivariate models were included in the multivariable model^(24)^. The linear trend test was performed for some variables by treating them as a continuous variable.

Based on the multivariable model and to quantify the proportion of childhood anaemia that could potentially be avoided if the population distribution of the associated factors was changed (all other factors remaining unchanged), we estimated the PAF, under the hypothetical assumption of a causal relationship. PAF were estimated only for the modifiable factors included in the model with PR > 1 to assess the possible public health implications of eliminating these factors towards childhood anaemia risk in the child population. Point and 95 % CI estimates of the PAF were calculated using the *punaf* command within the Stata software described by Newson^(25)^. The PAF estimate has the advantage of considering both the strength of the association between the risk factor and anaemia and the prevalence of that risk factor within the population.

Finally, age-stratified analyses were conducted to further explore age disparities in the associations and assess the robustness of the study results.

## Results

### Characteristics of study population

**Figure 1.**
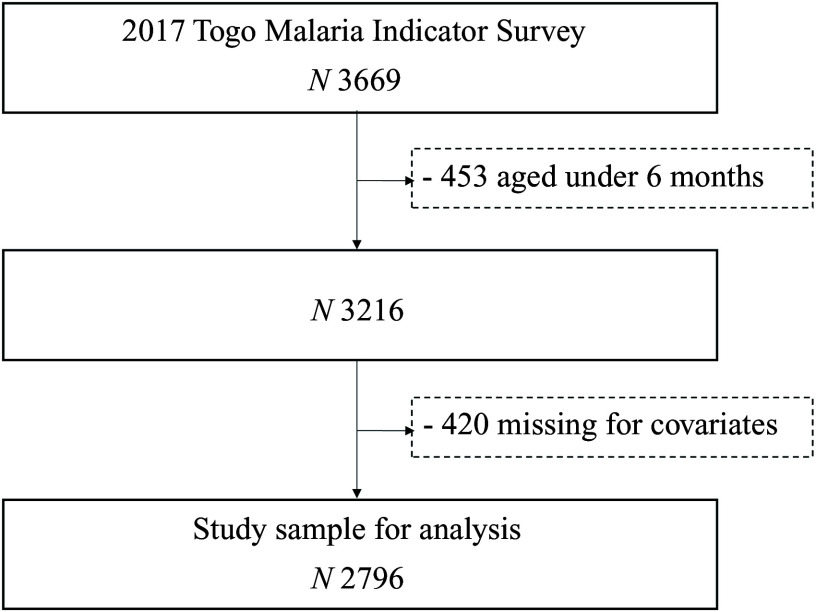
Participants flow diagram.

After excluding participants aged less than 6 months (*n* 453) and missing covariates (*n* 420), the study sample included 2796 participants (mean age: 31·2 ± 15·8; 50 % female) (Fig. 1). The characteristics of the eligible (*n* 2796) and excluded (*n* 420) participants are compared in the online Supplementary Table S1. Excluded participants did not differ from included participants in terms of sex, nutritional status, malaria infection, history of fever, maternal age, maternal educational level, the quality of the water source used and the type of sanitation facilities available but were significantly older (*P* < 0·0001). They were more likely to be the mother’s first or second child (*P* = 0·0001), be infected with malaria (*P* = 0·0125), have their mother anaemic (*P* = 0·0372), have an older head of household (*P* < 0·0001), live in a household with a high number of children 5 (*P* < 0·0001), live in middle-income household (*P* = 0·0002), live in a rural area (*P* = 0·0320) and live in Lomé and the Maritime and Central regions (*P* = < 0·0001). Table 1 provides the distribution of anaemia across childhood, maternal and household characteristics. The overall weighted prevalence of anaemia among children was 75·3 % (95 % CI 72·5, 78·0) with 76·6 % (73·8, 79·5) in male children and 73·9 % (70·2, 77·6) in female children. A total of 812 (29·0 %) children tested positive for malaria, while 697 (24·9 %) had had a recent episode of fever.

**Table 1.** Prevalence of anaemia among Togolese children aged 6–59 months by demographic, maternal and household characteristics. Malaria Indicator Survey, 2017 (Numbers and percentages; percentages and 95 % confidence intervals)

	Participants		Cases	Unweighted prevalence†		Weighted prevalence‡		*P*§
	*n*	%		%	95 % CI	%	95 % CI	
Total	2796	100·0	2118	75·8	73·5, 78·0	75·3	72·5, 78·0	
Child-related factors								
Sex								0·1319
Female	1398	50·0	1046	74·8	71·9, 77·7	73·9	70·2, 77·6	
Male	1398	50·0	1072	76·7	74·1, 79·3	76·6	73·8, 79·5	
Age (months)								< 0·0001
6–23	1032	36·9	895	86·7	84·3, 89·2	86·1	83·5, 88·7	
24–42	957	34·2	708	74·0	70·7, 77·2	74·7	70·8, 78·2	
43–59	807	28·9	515	63·8	59·9, 67·8	61·5	56·8, 66·1	
Birth order								0·0112
1–2	1133	40·5	817	72·1	69·1, 75·2	71·8	68·3, 75·4	
3	451	16·1	350	77·6	75·6, 81·7	75·7	70·6, 80·7	
≥ 4	1212	43·4	951	78·4	75·4, 81·5	78·6	74·8, 82·4	
Malnutrition								0·8072
Yes	160	5·7	119	74·4	67·6, 81·2	74·3	65·3, 83·2	
No	2636	97·3	1999	75·8	73·6, 78·1	75·3	72·7, 78·0	
Malaria infection								< 0·0001
Yes	812	29·0	725	89·3	87·1, 91·5	89·8	87·7, 92·0	
No	1984	71·0	1393	70·2	67·8, 72·6	69·8	66·7, 72·8	
History of fever (recent 2 weeks)								0·0075
Yes	697	24·9	569	81·6	78·2, 85·0	79·7	75·4, 84·0	
No	2099	75·1	1549	73·8	71·3, 76·2	73·7	70·9, 76·6	
Maternal factors								
Maternal age (years)								0·0332
≤ 25	828	29·6	649	78·4	75·3, 81·4	78·7	75·3, 82·1	
26–34	1273	45·5	963	75·6	72·5, 78·7	74·9	71·1, 78·7	
≥ 35	695	24·9	506	72·8	69·1, 76·5	71·8	67·5, 76·2	
Maternal anaemia*								< 0·0001
Yes	1405	50·3	1121	79·8	77·2, 82·4	80·2	77·5, 82·9	
No	1391	49·7	997	71·7	68·6, 74·7	70·0	66·1, 73·9	
Currently pregnant								0·3318
Yes	245	8·8	190	77·6	72·5, 82·6	78·1	72·0, 84·1	
No	2551	91·2	1928	75·6	73·3, 77·9	75·0	72·2, 77·8	
Maternal educational level								< 0·0001
No education	1252	44·8	1007	80·4	77·9, 83·0	80·3	77·5, 83·2	
Primary	894	32·0	667	74·6	71·3, 77·9	75·0	71·2, 78·7	
Secondary or higher	650	23·2	444	68·3	64·4, 72·2	67·9	63·5, 72·3	
Household factors								
Sex of household head								0·9749
Female	392	14·0	295	75·3	70·3, 80·2	75·2	69·8, 80·6	
Male	2404	86·0	1823	75·8	73·5, 78·1	75·3	72·4, 78·1	
Age of household head								0·4652
≤ 32	733	26·2	562	76·7	73·3, 80·1	77·0	73·2, 80·8	
33–48	1428	51·1	1082	75·8	73·1, 78·4	74·7	71·4, 78·1	
≥ 49	635	22·7	474	74·6	71·0, 78·3	74·2	69·8, 78·6	
Number of children 5 and under in household								0·0010
0–1	893	31·9	630	70·5	67·2, 73·9	71·0	67·0, 75·0	
2	1154	41·3	885	76·7	73·7, 79·7	76·2	72·8, 79·5	
≥ 3	749	26·8	603	80·5	77·0, 84·0	80·1	76·0, 84·1	
Household income status								< 0·0001
Low	1517	54·3	1218	80·3	77·7, 82·9	80·3	77·0, 83·6	
Middle	520	18·6	390	75·0	70·3, 79·7	76·7	71·9, 81·5	
High	759	27·1	510	67·2	63·3, 71·1	68·2	63·5, 72·8	
Unimproved drinking water source								0·0392
Yes	1030	36·8	816	79·2	75·6, 82·8	78·9	74·3, 83·5	
No	1766	63·2	1302	73·7	71·1, 76·3	73·4	70·4, 76·4	
Type of sanitation facilities								< 0·0001
Unimproved sanitation facilities	1855	66·3	1467	79·1	76·6, 81·6	79·1	75·9, 82·3	
Shared sanitation facilities	562	20·1	407	72·4	68·3, 76·5	72·2	68·0, 76·5	
Improved sanitation facilities	379	13·6	244	64·4	58·9, 69·9	66·4	60·2, 72·6	
Place of residence								0·0111
Rural	2108	75·4	1643	77·9	75·4, 80·5	77·8	74·7, 80·9	
Urban	688	24·6	475	69·0	64·8, 73·2	70·4	65·3, 75·5	
Region of residence								0·1989
Agglomeration of Lomé	374	13·4	272	72·7	67·2, 78·3	73·9	68·4, 79·3	
Maritime (without Lomé)	462	16·5	367	79·4	74·1, 84·8	77·3	71·0, 83·5	
Plateaux	507	18·1	407	80·3	73·8, 86·8	78·2	70·3, 86·1	
Centrale	388	13·9	276	71·1	65·2, 77·0	69·6	64·5, 74·8	
Kara	432	15·5	297	68·8	64·2, 73·3	69·2	64·9, 73·4	
Savanes	633	22·6	499	78·8	74·3, 83·4	78·0	73·4, 82·6	

*Hb < 110 g/L and Hb < 120 g/L for pregnant women. Low: 1st and 2nd quintiles; middle: 3rd quintile; high: 4th and 5th quintiles. †Crude estimates. ‡Survey weights were used to account for the unequal probability of selection of households induced by the sampling scheme as well as for non-response. §*P*-value of *χ* ^2^ Rao–Scott test.

### Assessment of factors associated with anaemia

Bivariate Poisson regression models showed significant associations with a *P*-value < 0·20 between potential risk factors and anaemia (Table 2). Factors associated with childhood anaemia in the bivariate models were the sex of child, age of child, birth order, malaria infection, fever status, history of fever in recent 2 weeks, maternal age, maternal anaemia, maternal educational level, number of children 5 and under in household, household income status, type of sanitation facilities, place of residence and region of residence.

**Table 2.** Bivariate and multivariable associations between explored risk factors and childhood anaemia and population-attributable fraction (PAF) (*n* 2796) (95 % confidence intervals)

	Anaemia (*n*+/*n*)	cPR	95 % CI	*P*	aPR	95 % CI	PAF (%)	95 % CI
Child-related modifiable factors							8·2	6·3, 10·1
Male sex	1072/1398	1·04	0·99, 1·09	0·144	1·03	0·98, 1·08	–	
Age (ref: 43–59 months)				< 0·001			–	
6–23	895/957	1·40	1·31, 1·50		1·46	1·37, 1·56	–	
24–42	708/807	1·22	1·12, 1·31		1·23	1·14, 1·32	–	
*P*-trend					< 0·001			
Birth order (ref: 1–2)				0·014			–	
3	350/451	1·05	0·98, 1·14		1·04	0·97, 1·12	–	
≥ 4	951/1212	1·09	1·03, 1·16		1·11	1·03, 1·19	–	
*P*-trend					0·005			
Malnutrition	119/160	0·99	0·88, 1·11	0·812			–	
Malaria infection‡	725/812	1·29	1·23, 1·35	< 0·001	1·30	1·22, 1·38	7·5	5·7, 9·2
History of fever (recent 2 weeks)‡	569/697	1·08	1·03, 1·14	0·004	1·03	0·98, 1·09	0·8	–0·6, 2·2
Maternal modifiable factors							11·1	5·7, 16·3
Mother’s age (ref: ≥ 35 years)				0·028			–	
≤ 25	649/828	1·10	1·02, 1·17		1·17	1·08, 1·27	–	
26–34	963/1273	1·04	0·98, 1·11		1·08	1·01, 1·15	–	
*P*-trend					< 0·001			
Maternal anaemia*‡	1121/1405	1·15	1·08, 1·21	< 0·001	1·13	1·07, 1·19	6·2	3·5, 8·9
Currently pregnant (ref: No or unsure)	190/245	1·04	0·96, 1·12	0·306	–		–	
Maternal educational level (ref: Secondary or higher)‡				< 0·001				
No education	1007/1252	1·18	1·10, 1·27		1·10	1·02, 1·18	3·9	1·0, 6·8
Primary	667/894	1·10	1·03, 1·18		1·04	0·98, 1·11	1·3	–0·7, 3·3
*P*-trend					0·003		–	
Household modifiable factors							15·8	8·6, 22·5
Male sex of household head	1823/2404	1·00	0·93, 1·08	0·975	–		–	
Age of household head (ref: ≥ 49 years)				0·454			–	
≤ 32	562/733	1·04	0·97, 1·11		–		–	
33–48	1082/1428	1·01	0·95, 1·07		–		–	
Number of children 5 and under in household (ref: 0–1)				0·004			–	
2	885/1154	1·07	1·01, 1·14		1·04	0·99, 1·10	–	
≥ 3	603/749	1·13	1·05, 1·21		1·07	1·00, 1·14	–	
*P*-trend					0·018			
Household income status (ref: High)‡				< 0·001				
Low	1218/1517	1·18	1·09, 1·28		1·16	1·04, 1·30	6·7	1·9, 11·3
Middle	390/520	1·13	1·03, 1·23		1·13	1·01, 1·26	2·2	0·2, 4·0
*P*-trend					0·006			
Unimproved drinking water source‡	816/1030	1·08	1·01, 1·15	0·035	0·96	0·91, 1·01	–	
Type of sanitation facilities (ref: Improved sanitation facilities)‡				0·001				
Unimproved sanitation facilities	1164/1855	1·19	1·08, 1·31		1·12	1·02, 1·22	6·5	1·2, 11·5
Shared sanitation facilities	407/562	1·09	0·98, 1·20		1·07	0·98, 1·17	1·3	–0·5, 3·1
*P*-trend					0·131			
Rural location (ref: Urban)†	1643/2108	1·11	1·02, 1·20	0·019	0·98	0·88, 1·10	–	
Region of residence (ref: Savanes)				0·035			–	
Agglomeration of Lomé	272/374	0·95	0·86, 1·04		1·28	1·14, 1·45	–	
Maritime (without Lomé)	367/462	0·99	0·90, 1·10		1·08	1·00, 1·16	–	
Plateaux	407/507	1·00	0·89, 1·13		1·01	0·93, 1·10	–	
Centrale†	276/388	0·89	0·81, 0·98		0·98	0·90, 1·05	–	
Kara†	297/432	0·89	0·81, 0·97		0·95	0·89, 1·01	–	
*P*-trend					0·011			
Modifiable overall factors							30·9	24·0, 37·2

*Hb < 110 g/L and Hb < 120 g/L for pregnant women. *n*, total number of children; *n*+, number of anaemia; cPR, crude prevalence ratio; aPR, adjusted prevalence ratio. *P*-value < 0·20 was significant in the bivariate models. ‡Modifiable factors. †prevalence ratio < 1 and the PAF was not calculated. Low: 1st and 2nd quintiles; middle: 3rd quintile; high: 4th and 5th quintiles. *P*-trend: *P*-value of a test for trend with a continuous variable across ordinal categories. PAF estimation model was adjusted for variables in multivariable models, including non-modifiable factors.

### Multivariable Poisson regression model and population-attributable fraction

Table 2 provides the results of the multivariable model and PAF at 5 % statistical significance. Factors related to the child that were significantly associated with anaemia were younger age (aPR = 1·46 (95 % CI = 1·37, 1·56) for the age group 6–23 months and aPR = 1·23 (1·14, 1·32) for 24–42 months compared with the age 43–59 months; *P*-trend < 0·001), a later birth order (aPR = 1·04 (0·97, 1·12) for a birth order 3 and 1·11 (1·03, 1·19) for a birth order 4 or higher compared with a birth order 1 or 2; *P*-trend = 0·005) and malaria infection (aPR = 1·30 (1·22, 1·38)). Maternal factors significantly associated with childhood anaemia were younger age (aPR = 1·17 (1·08, 1·27) for age ≤ 25 and 1·08 (1·01, 1·15) for age 26–34 compared with ≥ 35 years old; *P*-trend < 0·001), anaemia (aPR = 1·13 (1·07, 1·19)) and maternal educational level (aPR = 1·10 (1·02, 1·18) for no education and 1·04 (0·98, 1·11) for primary level compared with secondary level or higher; *P*-trend = 0·003). Regarding household factors, we found a significant increase in the risk of anaemia among children who lived with in a household with three or more children aged 5 years or younger compared with those who lived in a household with 0 or 1 child aged 5 years or younger, household income status (aPR = 1·16 (1·04, 1·30) for low income and aPR = 1·13 (1·01, 1·26) for middle income compared with high income; *P*-trend = 0·006), unimproved sanitation facilities (aPR = 1·12 (1·02, 1·22) compared with improved sanitation facilities) and region of residence (aPR = 1·28 (1·14, 1·45) for ‘agglomeration of Lomé’ compared with the ‘Savanes’ region).

After adjusting for non-modifiable factors significantly associated with childhood anaemia, the PAF of childhood anaemia cases for child-related factors were 7·5 % (95 % CI: 5·7, 9·2 %) for having malaria and 0·8 % (–0·6, 2·2 %) for recent episodes of fever (Table 2). For maternal risk factors, maternal anaemia explained 6·2 % (3·5, 8·9 %) of cases of childhood anaemia. The PAF associated with the educational level of the children’s mothers were 3·9 % (1·0, 6·8 %) for ‘no education’ and 1·3 % (–0·7, 3·3 %) for the ‘primary level’. Concerning the household factors, an estimated 6·7 % (1·9, 11·3 %) and 2·2 % (0·2, 4·0 %) of cases of childhood anaemia were attributable to low- and middle- household income, respectively, and 6·5 % (1·2, 11·5 %) for the use of unimproved sanitation facilities, while 1·3 % (–0·5, 3·1 %) of childhood anaemia cases were attributable to the use of shared sanitation facilities.

Overall, an estimated 8·2 % (95 % CI: 6·3, 10·1 %) of cases of childhood anaemia could be attributed to child-related modifiable factors, 11·1 % (5·7, 16·3 %) of cases to maternal-related ones, while 15·8 % (8·6, 22·5 %) of cases to household-related ones (Table 2). The overall estimated PAF for the combination of all modifiable factors was 30·9 % (24·0, 37·2 %).

### Sensitivity analyses

Results remained overall consistent in analyses stratified by age groups, especially in the 43–59 months age group where a stronger effect was observed compared with the other groups, especially for malaria infection and maternal educational level (online Supplementary Table S2). The overall PAF estimate for modifiable risk factors was highest for the 43–59-month group (48·2 % (28·2, 62·5 %)) compared with 30·3 % (18·7, 40·2 %) for 24–42 months and 19·9 % (10·7, 28·1 %) for 6–23 months (Table 2).

## Discussion

This nationwide study estimated the prevalence of anaemia in children aged 6–59 months and the proportion of cases of anaemia attributable to child-related, maternal and household factors in Togolese children aged 6–59 months. Similar to results from previous studies in Togo^(14)^ and other countries in SSA^(3,17,26)^, this study showed that about three in four children aged 6–59 months in Togo were suffering from anaemia. More significantly, our findings suggest that about 31 % childhood anaemia cases could potentially be avoided if exposure to modifiable risk factors were addressed at the population level, with notably greater benefits in children aged 43–59 months.

Prior studies examining different factors at different levels and their association with childhood anaemia have shown similar results as our findings. Our results are consistent with those of previous studies pooling individual data and examining different levels of factors associated with childhood anaemia^(12,17)^.

We estimated that 8·2 % of cases of childhood anaemia in Togo were attributable to child-related factors. Factors associated with childhood anaemia in this population have been described in the literature as iron deficiency, infection and poor feeding practices^(4–6)^. The fact that children aged 6–23 months had the highest risk for anaemia is probably due to the high demand for iron for growth and brain development at this age^(27)^ but also signifying the most vulnerable age group for childhood anaemia. In line with these findings, this study identified malaria infection as a factor associated with childhood anaemia with a PAF of 7·5 %. A study assessing factors associated with childhood anaemia in Ghana, Burkina Faso and Mali estimated a PAF of 15 % for malaria infection^(28)^, further highlighting the role of malaria in increasing the burden of childhood anaemia in the West African sub-region. Malaria is endemic in Togo with a prevalence of 28 % in children aged 6–59 months^(19)^. Anaemia is a known complication of malaria due to haemolysis of red blood cells, and over half of malaria-related deaths are attributable to severe anaemia (Hb concentration lower than 70 g/L)^(29–31)^. Prompt treatment of malaria, such as the promotion of breastfeeding in children under 5 years, could thus help reduce the burden of anaemia in this age group. In this respect, a recent cohort study in Brazil found that children who were breastfed for at least 12 months had a decreased risk for malaria in the first 2 years of life, despite *in utero* exposure to malaria^(32)^.

In this study, 11·1 % of cases of childhood anaemia could be attributed to maternal factors, in particular maternal anaemia. This is consistent with the results from a previous pooled study in SSA where 17 % of childhood anaemia was attributable to maternal factors^(17)^. Similar to previous studies^(33–35)^, young maternal age was associated with higher prevalence of childhood anaemia. Our finding of maternal anaemia as a significant attributable factor to childhood anaemia is supported by several studies^(14,17,36–38)^. The common environment shared by a mother and her child implies that they could have a mutual exposure to risk factors for anaemia. Shared physical, socio-economic, environmental and dietary risk factors may increase the risk for infectious diseases like gastrointestinal infections and helminthiases, which can predispose to anaemia^(39,40)^. This was also supported by our finding that a history of fever in the recent 2 weeks, signifying infections, is a contributing factor to childhood anaemia. Additionally, some evidence has suggested that anaemia during pregnancy increases the risk for childhood anaemia via low iron and ferritin levels^(41)^.

Babies born to mothers with iron deficiency are more likely to be iron deficient in early childhood with an associated risk of anaemia in childhood^(42)^. To reduce the risk of maternal anaemia, the WHO recommends iron supplementation for women of reproductive age in settings with prevalence of anaemia > 20 %^(43)^. However, adherence to iron supplementation during pregnancy has been highlighted as a problem in SSA, with a large population-based study reporting that less than one-third of pregnant women in Togo (27 %) adhered to iron supplementation for more than 90 days during their pregnancy^(44)^. Our findings suggest that efforts to address childhood anaemia should include efforts to prevent maternal anaemia, which may include improved adherence to iron supplementation initiatives.

In our study, children in a low-income household were 16 % more likely to be anaemic compared with a higher income household, and the highest proportion of attributable factors to childhood anaemia was due to household factors (15·8 %). Of these, low household income status and poor sanitary conditions were significant contributors. A low level of household income being a significant contributor to childhood anaemia in SSA has been reported elsewhere^(17,45)^. To the best of our knowledge, the PAF attributable to several household factors together has not been estimated. Evidence from studies in low-resource settings have found that a good household wealth index, which is often linked to household income status, is a protective factor for the occurrence of anaemia in children^(38,39,46)^. Initiatives to increase household incomes status could thus have a positive impact on reducing childhood anaemia burden in Togo. The association between household income and childhood anaemia could be mediated by nutritional deficiencies resulting from an inability to afford nutritious meals^(35)^. This explanation could also account for our finding of a greater number of children under the age of 5 years in the household being associated with increasing odds of childhood anaemia. Additionally, households with low income may find it difficult to access healthcare services for childhood illnesses^(45)^. In Togo, a recent assessment of the national health insurance scheme reported a positive association between household wealth and health-seeking behaviours, lending support to our findings^(47)^. Lack of money to buy nutritious foods can also result in nutritional deficiencies including iron deficiency and vitamin A, which have both been linked with childhood anaemia^(32)^. Poor sanitation facilities had a PAF of 6·5 % for childhood anaemia. The link between poor sanitation and anaemia has been hypothesised to be through two main pathways – intestinal infections^(40)^ and environmental enteropathy^(48)^ – which both contribute to decreased absorption of micronutrients necessary for the production of Hb. A recent meta-analysis of DHS and multiple indicator cluster surveys reported lower odds of anaemia among children living in a community with access to sanitation compared with those living in a community with less than 30 % access to sanitation^(49)^. Another meta-analysis including data from twenty-one countries reported a 12 % associated reduction in anaemia prevalence in children aged 6–59 months with access to improved household sanitation^(50)^.

This study has some limitations. The cross-sectional nature of our study design does not allow us to establish any causality, which is the assumption underlying the calculation of PAF. In addition, the cut-offs used to define some exposure levels may influence PAF estimates^(51)^ despite being chosen based on the literature and public health recommendations. Although PAF estimates are useful to rank risk factors, it is important to note that public health interventions are not possible theoretically for all factors, especially for non-modifiable risk factors such as age. Another limitation relates to responses collected during the 2017 TMIS that were self-reported and can be subject to recall bias. However, this is likely to be non-differential in nature and may not significantly skew the results though they may overestimate it. In addition, data on other risk factors such as iron supplementation, diet, children’s nutritional status and deworming, which may be confounding, were not available for adjustment and further analyses.

A major strength of this study is the nationally representative sample of children aged 6–59 months, and hence the results are externally valid. This is the first study of its kind using data from Togo to obtain population-based estimates of malaria indicators and also has a high statistical power. We were able to sort out and quantify the effects of various modifiable factors for childhood anaemia simultaneously in a single population and highlight factors with a greater potential impact on childhood anaemia. Additionally, the formula used to estimate the PAF from multivariable regression models allows for a non-biased estimation of adjusted PAF^(52)^. Finally, estimating the PAF is useful for comparing the population-level impacts of various risk factors of childhood anaemia. The PAF estimates provide useful information for the implementation of prevention programmes that target and prioritise the most impactful modifiable risk factors for more effective interventions to reduce the health, economic and social impacts of childhood anaemia nationwide.

### Conclusion

This study highlighted a high prevalence of anaemia among children aged from 6 to 59 months in Togo and the multifactorial nature of the associated risk factors, suggesting that interventions addressing childhood anaemia need to be integrated to address child-related, maternal and household factors. The importance of child-related (including malaria and fever), maternal (including anaemia and educational level) and household-related (including income status and the presence of sanitation facilities) modifiable factors were highlighted in the present study to aid appropriate population-level interventions to address the significant burden of childhood anaemia in Togo.

## Supporting information

Ohene-Agyei et al. supplementary material Ohene-Agyei et al. supplementary material

## Supplementary material

For supplementary material/s referred to in this article, please visit https://doi.org/10.1017/S0007114524003313.
