# Supplementary material for: Population-attributable fractions for risk factors for childhood anaemia: findings from the 2017 Togo Malaria Indicator Survey
Source: Br J Nutr. 2025 Jan 16;134(9):764–72. doi: 10.1017/S0007114524003313 (PMC12766107; doi:10.1017/S0007114524003313)
Supplement: Ohene-Agyei et al. supplementary material [file S0007114524003313sup001.docx]

**Supplementary Material:**

**Potentially preventable proportion of childhood anaemia: Findings from the 2017 Togo Malaria Indicator Survey**

Phyllis Ohene-Agyei^1,2,3#^, Aude Laetitia Ndoadoumgue^1,3#^, Essossimna Bana-Ewai, MD^1,4,5*^, Issifou Yaya^1,3,6^, Aboubakari Nambiema^1,3,7^

Corresponding author: Essossimna Bana-Ewai, MD

Unité de Recherche en Santé des Populations (URESAP), CHU SO, Lomé, Togo.

Email: [banaeg@gmail.com](mailto:banaeg@gmail.com)

**Table S1. Comparison of the characteristics of the eligible and excluded participants**

|  | **Eligible participants (N=2796)** | **Excluded participants (N=420)** | ***P-value***† |
| --- | --- | --- | --- |
| Child related factors | | | |
| Sex |  |  | 0.7159 |
| Female | 50.0 | 51.0 |  |
| Male | 50.0 | 49.0 |  |
| Age (months) |  |  | <.0001 |
| 6-23 | 36.9 | 13.8 |  |
| 24-42 | 34.2 | 39.3 |  |
| 43-59 | 28.9 | 46.9 |  |
| Birth order |  |  | 0.0125 |
| 1-2 | 40.5 | 52.1 |  |
| 3 | 16.1 | 21.9 |  |
| ≥4 | 43.3 | 26.0 |  |
| Malnutrition | 5.7 | 4.1 | 0.5568 |
| Malaria infection | 29.0 | 33.7 | 0.0502 |
| History of fever (recent 2 weeks) | 24.9 | 29.0 | 0.4421 |
| Maternal factors | | | |
| Maternal age (years) |  |  | 0.3958 |
| ≤25 | 29.6 | 37.0 |  |
| 26-34 | 45.5 | 41.1 |  |
| ≥35 | 24.9 | 21.9 |  |
| Maternal anaemia* | 50.3 | 62.9 | 0.0372 |
| Currently pregnant | 8.8 | 13.7 | 0.1435 |
| Maternal educational level |  |  | 0.1013 |
| No education | 44.8 | 37.0 |  |
| Primary | 32.0 | 43.8 |  |
| Secondary or higher | 23.2 | 19.2 |  |
| Household factors | | | |
| Sex of household head |  |  | <.0001 |
| Female | 14.0 | 31.5 |  |
| Male | 86.0 | 68.5 |  |
| Age of household head |  |  | <.0001 |
| ≤32 | 26.2 | 12.3 |  |
| 33-48 | 51.1 | 35.6 |  |
| ≥49 | 22.7 | 52.1 |  |
| Number of children five and under in household |  |  | <.0001 |
| 0-1 | 31.9 | 67.1 |  |
| 2 | 41.3 | 13.7 |  |
| ≥3 | 26.8 | 19.2 |  |
| Household income status |  |  | 0.0002 |
| Low | 54.3 | 55.2 |  |
| Middle | 18.6 | 25.5 |  |
| High | 27.1 | 19.3 |  |
| Improved drinking water source | 63.2 | 77.8 | 0.4996# |
| Type of sanitation facilities |  |  | 0.7706# |
| Unimproved sanitation facilities | 66.3 | 77.8 |  |
| Shared sanitation facilities | 20.1 | 22.2 |  |
| Improved sanitation facilities | 13.6 | 0.0 |  |
| Place of residence |  |  | 0.0320 |
| Rural | 24.6 | 13.7 |  |
| Urban | 75.4 | 86.3 |  |
| Region of residence |  |  | <.0001 |
| Agglomeration of Lomé | 13.4 | 6.8 |  |
| Maritime (without Lomé) | 16.5 | 37.0 |  |
| Plateaux | 18.1 | 13.7 |  |
| Centrale | 13.9 | 32.9 |  |
| Kara | 15.5 | 1.4 |  |
| Savanes | 22.6 | 8.2 |  |
| *****Hb<11.0 g/dl and Hb<12.0 g/dl for pregnant women. Low: 1^st^ and 2^nd^ quintiles, middle: 3^rd^ quintile, High: 4^th^ and 5^th^ quintiles. The results are presented as percentages. †p-value of Chi-Square test for categorical variables. #Fisher's exact test. | | | |

**Table S2. Age stratified multivariable associations between explored risk factors and childhood anaemia, and population-attributable fraction (PAF)**

|  |  | **6-23 months of age** | |  | **24-42 months of age** | |  | **43-59 months of age** | |  |
| --- | --- | --- | --- | --- | --- | --- | --- | --- | --- | --- |
|  | **Prevalence** | **aPR (95% CI)** | **PAF (%) (95% CI)** | **Prevalence** | **aPR (95% CI)** | **PAF (%) (95% CI)** | **Prevalence** | **aPR (95% CI)** | **PAF (%) (95% CI)** |  |
| Child-related modifiable factors |  |  | 3.8 (1.6; 5.9) |  |  | 8.4 (4.9; 11.7) |  |  | 15.5 (10.8; 20.0) |  |
| Male sex | 49.2 | 1.04 (0.98; 1.11) | - | 52.2 | 1.06 (0.98; 1.15) | - | 48.3 | 0.99 (0.89; 1.11) | - |  |
| Birth order (ref: 1-2) |  |  | - |  |  | - |  |  | - |  |
| 3 | 16.7 | 1.04 (0.95; 1.13) | - | 16.5 | 1.06 (0.94; 1.20) | - | 15.0 | 1.05 (0.87; 1.27) | - |  |
| ≥4 | 42.5 | 1.03 (0.96; 1.12) | - | 42.6 | 1.18 (1.06; 1.31) | - | 45.2 | 1.18 (1.01; 1.39) | - |  |
| Malaria infection‡ | 20.7 | 1.11 (1.04; 1.19) | 3.0 (1.2; 4.8) | 29.5 | 1.27 (1.15; 1.40) | 7.2 (4.3; 10.0) | 39.2 | 1.58 (1.39; 1.80) | 14.4 (10.3; 18.3) |  |
| History of fever (recent 2 weeks)‡ | 26.0 | 1.03 (0.97; 1.09) | 0.8 (-0.7; 2.3) | 26.1 | 1.05 (0.97; 1.14) | 1.3 (-1.0; 3.5) | 22.2 | 1.05 (0.93; 1.19) | 1.3 (-2.2; 4.7) |  |
| Maternal modifiable factors |  |  | 7.4 (0.6; 13.8) |  |  | 11.5 (2.1; 20.0) |  |  | 16.2 (1.8; 28.5) |  |
| Mother's age (ref: ≥35 years) |  |  | - |  |  | - |  |  | - |  |
| ≤25 | 36.2 | 1.07 (0.96; 1.18) | - | 29.0 | 1.16 (1.01; 1.34) | - | 21.8 | 1.44 (1.18; 1.75) | - |  |
| 26-34 | 44.4 | 1.04 (0.95; 1.14) | - | 45.4 | 1.04 (0.93; 1.16) | - | 47.2 | 1.21 (1.05; 1.39) | - |  |
| Maternal anaemia*‡ | 48.6 | 1.10 (1.02; 1.17) | 4.7 (1.2; 8.1) | 51.2 | 1.19 (1.10; 1.28) | 8.9 (5.1; 12.7) | 51.2 | 1.13 (1.00; 1.29) | 6.5 (-0.3; 12.9) |  |
| Maternal educational level (ref: Secondary or higher)‡ |  |  |  |  |  |  |  |  |  |  |
| No education | 40.7 | 1.05 (0.97; 1.13) | 1.8 (-1.4; 5.0) | 47.3 | 1.06 (0.93; 1.21) | 2.4 (-3.3; 7.7) | 47.0 | 1.22 (1.01; 1.47) | 8.5 (0.7; 15.7) |  |
| Primary | 32.3 | 1.03 (0.96; 1.11) | 1.1 (-1.4; 3.4) | 32.8 | 1.01 (0.89; 1.15) | 0.4 (-3.7; 4.4) | 30.6 | 1.06 (0.88; 1.28) | 1.9 (-4.0; 7.5) |  |
| Household modifiable factors |  |  | 10.2 (1.0; 18.6) |  |  | 14.6 (3.6; 24.2) |  |  | 28.1 (4.2; 46.0) |  |
| Number of children 5 and under in household (ref: 0-1) |  |  | - |  |  | - |  |  | - |  |
| 2 | 33.6 | 1.00 (0.95; 1.07) | - | 38.5 | 1.13 (1.01; 1.26) | - | 45.0 | 1.03 (0.89; 1.18) | - |  |
| ≥3 | 41.0 | 0.96 (0.87; 1.04) | - | 28.7 | 1.22 (1.10; 1.37) | - | 26.3 | 1.07 (0.92; 1.23) | - |  |
| Household income status (ref: High)‡ |  |  |  |  |  |  |  |  |  |  |
| Low | 53.7 | 1.16 (1.03; 1.31) | 6.6 (1.6; 11.2) | 54.1 | 1.15 (0.94; 1.41) | 6.3 (-2.4; 14.4) | 55.1 | 1.19 (0.90; 1.57) | 8.1 (-5.2; 19.7) |  |
| Middle | 18.4 | 1.10 (0.99; 1.22) | 1.7 (-0.1; 3.5) | 18.8 | 1.17 (0.98; 1.40) | 2.8 (-0.3; 5.8) | 18.6 | 1.13 (0.87; 1.45) | 2.2 (-2.5; 6.6) |  |
| Unimproved drinking water source‡ | 39.1 | 0.96 (0.90; 1.02) | - | 34.5 | 0.96 (0.88; 1.05) | - | 36.8 | 0.97 (0.85; 1.11) | - |  |
| Type of sanitation facilities (ref: Improved sanitation facilities)‡ |  |  |  |  |  |  |  |  |  |  |
| Unimproved sanitation facilities | 67.3 | 1.03 (0.92; 1.15) | 1.8 (-4.9; 8.0) | 66.4 | 1.12 (0.96; 1.31) | 6.7 (-2.7; 15.3) | 65.1 | 1.28 (0.90; 1.82) | 14.4 (-5.9; 30.9) |  |
| Shared sanitation facilities | 18.5 | 1.02 (0.91; 1.15) | 0.4 (-2.0; 2.8) | 20.9 | 0.97 (0.84; 1.14) | - | 21.2 | 1.36 (0.97; 1.89) | 5.7 (-0.4; 11.4) |  |
| Rural location (ref: Urban)† | 73.9 | 0.93 (0.84; 1.02) | - | 76.1 | 1.02 (0.81; 1.29) | - | 76.5 | 1.15 (0.84; 1.59) | - |  |
| Region of residence (ref: Savanes) |  |  | - |  |  | - |  |  | - |  |
| Agglomeration of Lomé | 13.8 | 1.08 (0.95; 1.22) | - | 12.9 | 1.47 (1.16; 1.86) | - | 13.5 | 1.62 (1.13; 2.31) | - |  |
| Maritime (without Lomé) | 15.5 | 1.01 (0.91; 1.11) | - | 16.4 | 1.07 (0.92; 1.24) | - | 18.0 | 1.19 (1.03; 1.37) | - |  |
| Plateaux | 18.5 | 0.99 (0.90; 1.09) | - | 18.4 | 1.06 (0.93; 1.21) | - | 17.3 | 0.98 (0.85; 1.14) | - |  |
| Centrale† | 14.6 | 1.00 (0.90; 1.12) | - | 14.1 | 0.91 (0.79; 1.05) | - | 12.6 | 0.99 (0.81; 1.23) | - |  |
| Kara† | 15.1 | 1.02 (0.94; 1.10) | - | 16.0 | 0.86 (0.73; 1.01) | - | 15.2 | 1.00 (0.87; 1.16) | - |  |
| Modifiable overall factors |  |  | 19.9 (10.7; 28.1) |  |  | 30.3 (18.7; 40.2) |  |  | 48.2 (28.2; 62.5) |  |
| *Hb<11.0 g/dl and Hb<12.0 g/dl for pregnant women. aPR: adjusted prevalence ratio. ‡Modifiable factors. PAF: Population-attributable fraction. 95% CI: 95% confidence interval. †prevalence ratio<1 and the PAF was not calculated. Low: 1^st^ and 2^nd^ quintiles, middle: 3^rd^ quintile, High: 4^th^ and 5^th^ quintiles. All PAF estimation models were adjusted for variables in multivariable models, including non-modifiable factors. | | | | | | | | | | |
